# A novel thermostable d-amino acid oxidase of the thermophilic fungus *Rasamsonia emersonii* strain YA

**DOI:** 10.1038/s41598-019-48480-y

**Published:** 2019-08-16

**Authors:** Yuya Shimekake, Takehiro Furuichi, Katsumasa Abe, Yoshio Kera, Shouji Takahashi

**Affiliations:** Department of Bioengineering, Nagaoka University of Technology, Nagaoka, Niigata 940-2188 Japan

**Keywords:** Protein purification, Biomaterials - proteins

## Abstract

d-Amino acid oxidase (DAAO) is a valuable flavoenzyme capable of being used in various practical applications, such as in determining d-amino acids and producing a material for semisynthetic cephalosporins, requiring higher thermal stability, higher catalytic activity, and broad substrate specificity. In this study, we isolated the thermophilic fungus *Rasamsonia emersonii* strain YA, which can grow on several d-amino acids as the sole nitrogen source, from a compost and characterized DAAO (ReDAAO) of the fungus. ReDAAO expressed in *Escherichia coli* exhibited significant oxidase activity against various neutral and basic d-amino acids, in particular hydrophobic d-amino acids. In addition, the enzyme also significantly acted on cephalosporin C, a starting material for semisynthetic antibiotics, and d-Glu, a general substrate for d-aspartate oxidase but not for DAAO, showing its unique and practically useful substrate specificity. The apparent *k*_cat_ and *K*_m_ values of the enzyme toward good substrates were comparable to those of higher catalytic fungal DAAOs, and the thermal stability (*T*_50_ value of ~60 °C) was comparable to that of a thermophilic bacterial DAAO and significantly higher than that of other eukaryotic DAAOs. These results highlight the great potential of ReDAAO for use in practical applications.

## Introduction

d-Amino acid oxidase (DAAO, EC 1.4.3.3) catalyzes the oxidative deamination of neutral and basic d-amino acids with flavin adenine dinucleotide (FAD) as a cofactor. The same reaction of acidic d-amino acids is catalyzed by another flavin enzyme, d-aspartate oxidase (DDO, EC 1.4.3.1). DAAO was first found in the pig kidney and, subsequently, in various eukaryotic organisms as a peroxisomal protein and a part of prokaryotic organisms^1^. In eukaryotic organisms, DAAO has been known to play a role as a detoxifying reagent in the metabolic degradation of exogenous and endogenous d-amino acids^2^. It has also been reported to be responsible for helping to utilize d-amino acids as nutritional and energy sources for cell growth in fungi^3^. In the human brain, DAAO is suggested to be involved in the central nervous system and its related disorders, such as schizophrenia and amyotrophic lateral sclerosis (ALS), through the metabolism of d-Ser, which is a co-agonist of the *N*-methyl d-aspartate receptor^4–7^. Decreased and increased levels of d-Ser have been observed in schizophrenia and ALS patients, respectively^8,9^, suggesting the possibility of an early diagnosis of these diseases by measuring d-Ser levels *in vivo*.

DAAO is an enzyme that is valuable for use in various applications^10–12^, such as the optical resolution of a racemic mixture of amino acids^13^; detection and quantification of d-amino acids^14^; and production of α-keto acids, which can be used in pharmaceutical materials^15^. In particular, the use of this enzyme is most important in the production of 7-aminocephalosporanic acid (7-ACA), an initial material of approximately 50 types of β-lactam antibiotics, from cephalosporin C (CPC)^12^. DAAOs of the mesophilic fungi *Rhodotorula gracilis* (RgDAAO) and *Trigonopsis variabilis* (TvDAAO) have been known to be useful in several practical applications because of their high catalytic activity and broad substrate specificity^16^; however, their poor stability inhibits application over a wide spectrum. To obtain a highly thermostable DAAO, we have previously identified a DAAO homologous gene in the thermophilic bacterium *Rubrobacter xylanophilus* (*RxDAAO*), and the recombinant RxDAAO has been shown to have the highest thermal stability among the known DAAOs^17^; however, the substrate specificity of this thermophilic bacterial enzyme is narrow and limited to branched-chain d-amino acids, such as d-Val and d-Ile. Moreover, the catalytic activity is much lower than those of the fungal RgDAAO and TvDAAO.

Thermophilic fungi have been an attractive source of industrially useful enzymes, such as lipase, protease, xylanase, and cellulase, because enzymes from thermophilic fungi are usually highly stable^18^. We have also recently reported that DDO from the thermophilic fungus *Thermomyces dupontii* NBRC 30541 has high thermal stability than that of other DDOs and high catalytic activity^19^. However, DAAO has not been identified and isolated from thermophilic fungi. One of thermophilic fungi, *Rasamsonia emersonii* (phylum, Ascomycota), has been found in composts^20^ and grows well at 45–50 °C^21^. This fungus also produces several biotechnologically valuable enzymes with high thermal stability, such as β-glucosidase, α-glucuronidase, and FAD-dependent glucose dehydrogenase^20,22,23^, suggesting it to be a promising source of various thermostable enzymes including DAAO.

In this study, we isolated the thermophilic fungus *R*. *emersonii* strain YA that can utilize d-amino acids for cell growth from a compost and isolated a DAAO homologous gene of the fungus. The gene product (ReDAAO) expressed in *Escherichia coli* exhibited oxidase activity against various neutral and basic d-amino acids. The enzyme also significantly acted on CPC and, interestingly, an acidic d-amino acid, d-Glu, which is a general substrate for DDO but not for DAAO. Additionally, the enzyme showed higher catalytic activity than most bacterial and animal DAAOs and higher thermal stability than that of eukaryotic DAAOs. Altogether, ReDAAO, which was the first characterized thermophilic fungal DAAO, showed unique broad substrate specificity and more advantageous properties for practical applications than other DAAOs.

## Results

### Isolation of thermophilic fungi that can utilize d-amino acids for cell growth

**Figure 1:**
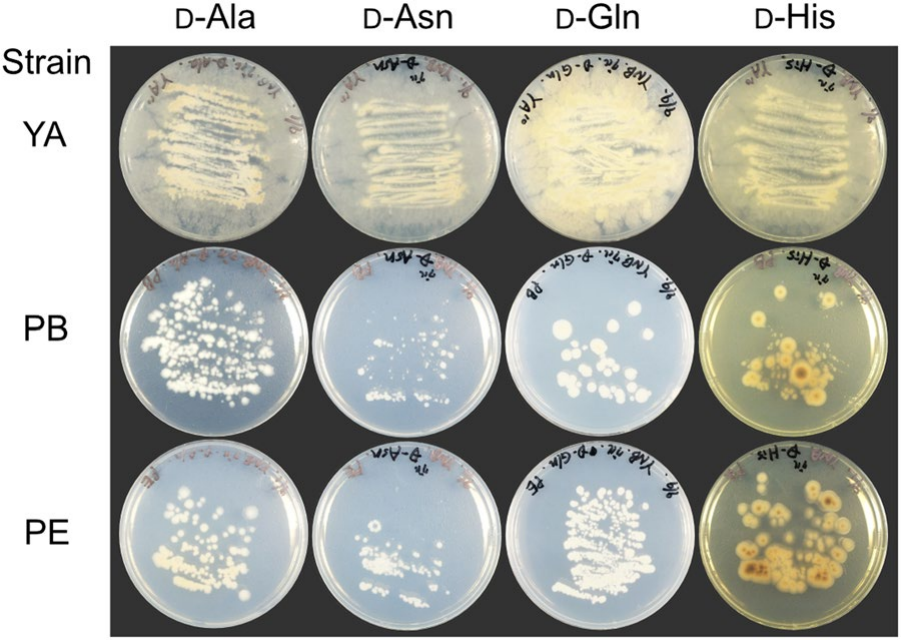
d-Amino acid-assimilating ability of isolated thermophilic fungi. The strains were cultivated at 50 °C for 1 week on a minimal medium containing 50 mM d-Ala, d-Asn, d-His, or d-Gln as the sole nitrogen source.

To isolate thermophilic fungi, we inoculated a suspension of various composts onto PDA medium and cultivated them at 50 °C and 60 °C for 2 weeks. Three fungi were obtained at 50 °C from bark and cattle manure composts—strain YA and strains PB and PE, respectively; whereas, no fungi growth was observed at 60 °C. These thermophilic fungi were able to grow on a minimal medium containing d-Ala, d-Asn, d-Gln, or d-His as the sole nitrogen source, which are better substrates of broad substrate specificity DAAOs such as RgDAAO and TvDAAO^16^ (Fig. 1). In particular, strain YA grew better on all the d-amino acids, especially on d-Gln, than the other strains. The nucleotide sequence of the ITS region of strain YA showed a high sequence identity with that of the thermophilic fungi *R*. *emersonii* strain CBS 393.64 (99.7%) and strain CBS 396.64 (100%); which suggests that it belongs to *R*. *emersonii* and to which we assigned it (Supplementary Fig. 1).

### Identification of DAAO homologous gene of strain YA

**Figure 2:**
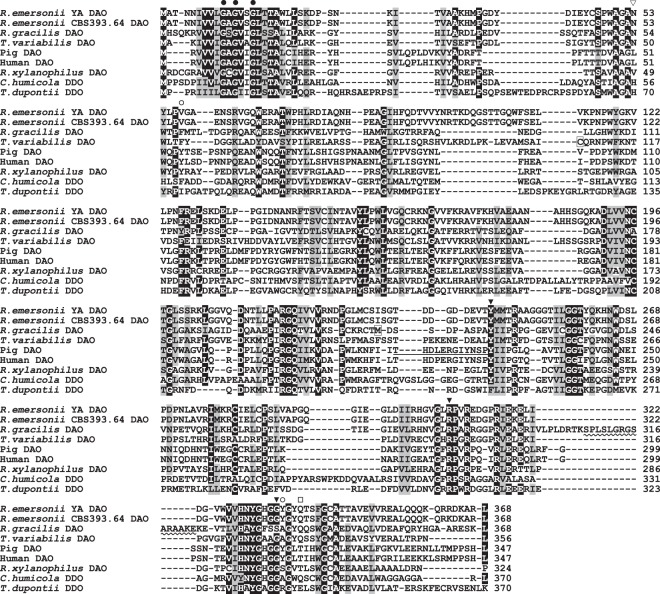
Multiple sequence alignment of ReDAAO with those of other known DAAOs and DDOs. DAAOs of *Rhodotorula gracilis* (Swiss-Prot, P80324), *Trigonopsis variabilis* (GenBank, CAA90322), pig kidney (GenBank, NP_999231), human (GenBank, NP_001908), and *Rubrobacter xylanophilus* (GenBank, BAP18969). DDOs of *Cryptococcus humicola* (GenBank, BAD13387) and *Thermomyces dupontii* (Fungal Genomes, Talth1p4_002474). The closed circles show the nucleotide-binding motif. The open circles indicate the position of the gate-anchoring residues, Tyr55 and Tyr314, in mammalian DAAOs. The wavy line shows the dimerization loop of RgDAAO. The open triangle shows the amino acid residues conserved in fungal DAAOs (Asn residue) and DDOs (His residue). The closed triangles show the amino acid residues interacting with the α-carboxy and α-amino groups of the substrates. The open square indicates the position of Gln residue that fixes the α-amino groups of substrates through a water molecule. The active site lid of pkDAAO is underlined. Met213 of RgDAAO and Cys108 of TvDAAO are in squares.

To identify the DAAO homologous gene of strain YA, we first searched for the gene encoding putative DAAO protein in the genome sequence of a type strain of *R*. *emersonii* strain CBS 393.64 using the amino acid sequence of fungal DAAOs. The search gave a gene (*T310_5354*) that is annotated to code for putative DAAO. The genomic gene was predicted to comprise six exons spanning 1,443 bp and to encode a protein of 368 amino acids (Fig. 2). Based on the nucleotide sequence of strain CBS 393.64, we obtained a DAAO homologous gene of strain YA using PCR. The nucleotide sequence and the exon-intron structure of the strain YA homologous gene was identical to those of strain CBS 393.64 except for one base (G instead of A in the gene of strain CBS 393.64) near the 3′-end in the ORF, which causes a difference in the amino acid sequence as follows: Arg363 in strain YA DAAO homolog instead of Lys363 in strain CBS 393.64 DAAO (Fig. 2).

In the amino acid sequence of the putative DAAO of strain YA (ReDAAO), a nucleotide-binding motif (GXGXXG, where “X” is any amino acid) was observed in the N-terminal region (Fig. 2). The Tyr and Arg residues that interact with the α-carboxy group of the substrates in DAAOs were conserved at positions 244 and 308 of ReDAAO, respectively, and the Gly residue that interacts with the α-amino group of the substrates was found at position 335. An Asn and a Gln residues that are conserved in the active site of fungal DAAOs and fixe the α-amino group of substrates via hydrogen bonding through a water molecule in RgDAAO were found at positions 53 and 339, respectively^19,24^. In the corresponding position of Met213 of RgDAAO, which plays an important role in the substrate specificity of RgDAAO^25^, an Ile residue was present at position 232. A consensus type-1 peroxisome-targeting consensus sequence [STAGCN]-[RKH]-[LIVMAFY] (PROSITE, PS00342) was found in the C-terminus. These findings in the amino acid sequence suggested that *ReDAAO* gene could encode functional DAAO.

### Expression in *E*. *coli* and purification of ReDAAO

**Figure 3:**
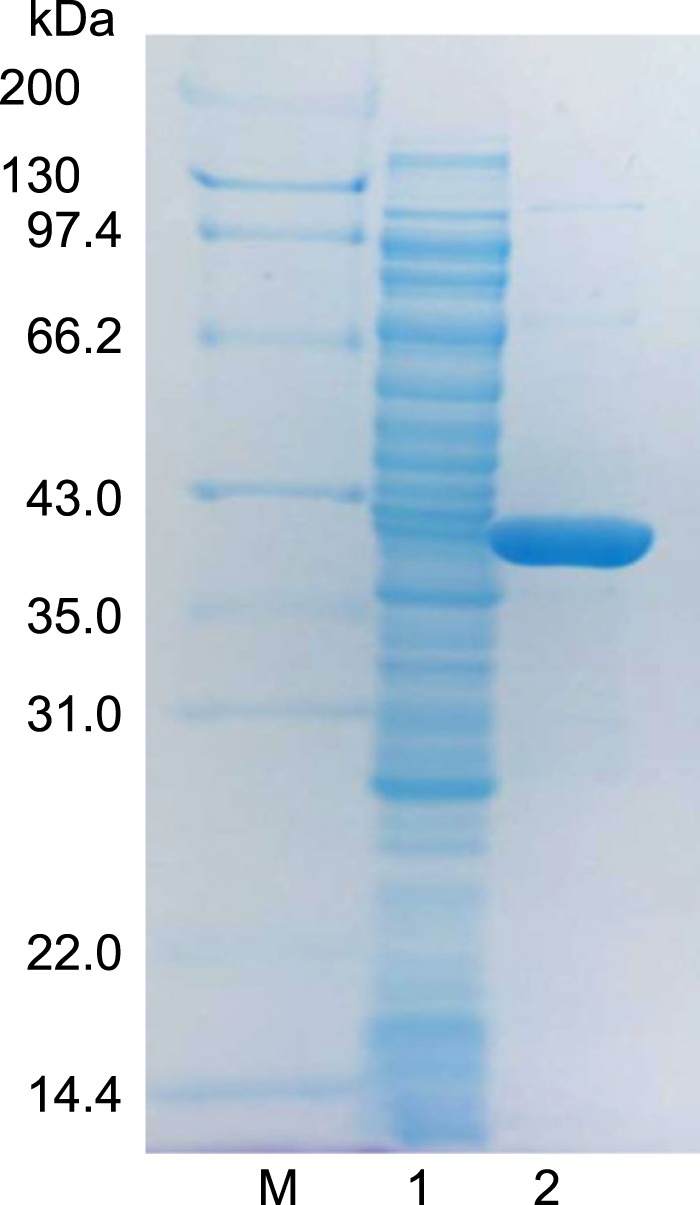
SDS-PAGE of purified ReDAAO. Lanes: M, protein marker; 1, crude extract; 2, purified ReDAAO. The original uncropped gel image is shown in Supplementary Fig. 3.

To confirm that *ReDAAO* gene encodes functional DAAO, ReDAAO was expressed in *E*. *coli* as a histidine-tagged fusion protein. The recombinant protein was efficiently expressed as a soluble protein in *E*. *coli* and was estimated to account for ~2% of the soluble proteins in the crude extract using SDS-PAGE (Fig. 3). The crude extract exhibited oxidase activity against neutral and basic d-amino acids, in particular d-Ile and d-Val (Supplementary Table 1). Interestingly, the crude extract also exhibited a significant activity against an acidic d-amino acid, d-Glu, a general substrate of DDO but not of DAAO. ReDAAO was purified approximately 25-fold with a yield of 25.7% from the crude extract, which gave 1.43 mg purified enzyme from 600 ml culture broth (Supplementary Table 2). The purified protein migrated as a single band with a molecular mass of 42 kDa using SDS-PAGE (Fig. 3) and 185 kDa, both at 0.01 and 0.1 mg/ml protein concentrations, using a gel filtration column, which suggested that the enzyme is a tetramer. The purified ReDAAO showed a specific activity of 70.0 and 112 U/mg at 37 °C and 55 °C, respectively, toward d-Ile as a substrate. The results suggested that the recombinant protein is DAAO with broad substrate specificity and high catalytic activity.

### Cofactor analyses

**Figure 4:**
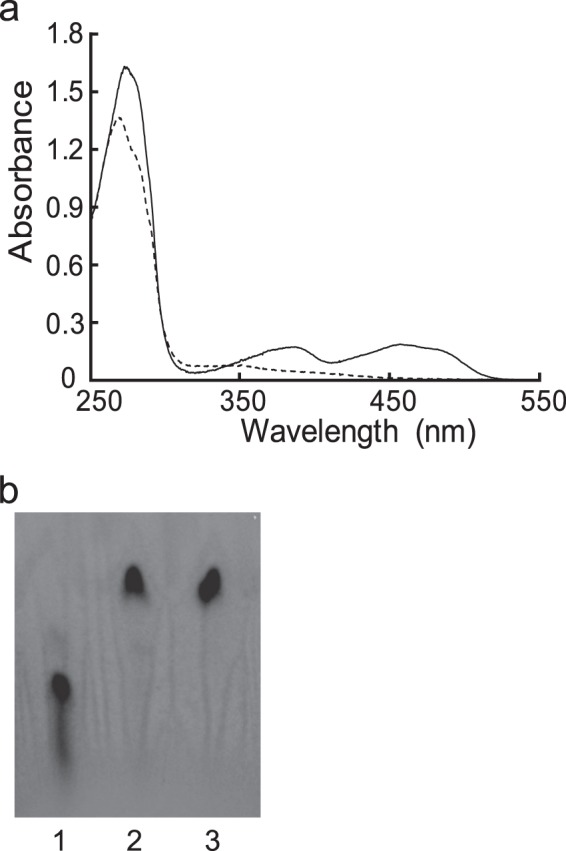
Cofactor analysis of ReDAAO. (**a**) Absorption spectra of ReDAAO. 20 μM ReDAAO was analyzed before (solid line) and after (dashed line) incubating with 20 mM d-Ile at 40 °C for 10 min. (**b**) TLC analysis of ReDAAO flavin. Lanes: 1. 0.1 mM FMN; 2, 0.1 mM FAD; 3, flavin from 0.185 mM ReDAAO. The original uncropped TLC image is shown in Supplementary Fig. 4.

To determine whether ReDAAO contains flavin, we analyzed its absorption spectrum (Fig. 4a). The protein solution was yellow and exhibited a typical absorption spectrum of flavoproteins, with maximum absorption intensities of 273, 370, and 455 nm. The maximum visible absorption disappeared by adding d-Ile to the protein solution. After removing the thermally denatured protein, the supernatant was also yellow and within the flavin absorption spectrum (data not shown), which indicated a noncovalent association between flavin and the protein. The TLC analysis of the supernatant showed a single fluorescent spot that was identical to that of the FAD standard, suggesting that the prosthetic group could be FAD (Fig. 4b). These results suggested that ReDAAO contains noncovalently bound FAD that functions as a cofactor.

### Substrate specificity and kinetic parameters

We analyzed the substrate specificity of purified ReDAAO using various d-amino acids, some l-amino acids, and CPC as substrates (Table 1). The enzyme showed high activities against various d-amino acids, especially d-Val and d-Ile, but not against the corresponding l-isomers. As in the case of the crude extract, the purified enzyme also exhibited significant oxidase activities toward d-Glu as well as CPC, which were approximately 56% and 14%, respectively, of that against d-Val, the best substrate. These results revealed that ReDAAO might have novel broad substrate specificity and might be potentially valuable for practical applications.

**Table 1:** Substrate specificity of purified ReDAAO.

Substrates	Mean relative activity (%) ± SD
d-Val	100
d-Ile	88.4 ± 5.3
d-Phe	82.3 ± 9.6
d-Met	74.2 ± 2.3
d-Ala	70.0 ± 15.9
d-Leu	64.6 ± 5.3
d-Gln	60.5 ± 11.8
d-*allo*-Thr	53.2 ± 2.2
d-Glu	53.0 ± 0.8
d-Arg	45.9 ± 2.0
d-*allo*-Ile	44.6 ± 2.5
d-Ser	41.3 ± 4.7
d-His	30.6 ± 3.8
d-Tyr	30.4 ± 0.8
d-Asn	27.6 ± 0.7
d-Trp	17.3 ± 3.8
d-Thr	16.1 ± 1.1
d-Lys	12.6 ± 1.4
d-Pro	5.2 ± 0.4
d-Asp	2.5 ± 0.1
CPC	14.1 ± 0.5
Gly	0.9 ± 0.7
l-Ile	nd
l-Val	nd
l-Met	nd

The enzyme activity was measured using the HRP-coupled method with 20 mM each amino acid except 2 mM d-Tyr and 1 mM cephalosporin C (CPC) at 37 °C. Each data represents the mean ± SD of triplicate measurements. nd indicates not detected.

Next, we determined the kinetic parameters of ReDAAO for some neutral d-amino acids as well as for d-Glu and d-Arg (Table 2). The enzyme exhibited typical Michaelis–Menten kinetics for each substrate (Supplementary Fig. 2). The highest apparent catalytic rate constant (*k*_cat_) was observed for d-Val, followed by d-Ala, d-Met, d-Glu, and d-Arg, in decreasing order, and the lowest apparent Michaelis constant (*K*_m_) was obtained for d-Met, followed by d-Val, d-Ala, d-Glu, and d-Arg, in increasing order, thus, providing the highest apparent catalytic efficiency (*k*_cat_/*K*_m_) for d-Met followed by d-Val, d-Ala, d-Glu, and d-Arg, in decreasing order.

**Table 2:** Kinetic parameters of purified ReDAAO.

Substrates	*k*_cat_ (s^−1^)	*K*_m_ (mM)	*k*_cat_/*K*_m_ (s^−1^ M^−1^)
d-Met	120 ± 3.1	0.213 ± 0.03	562,000
d-Val	225 ± 2.4	0.628 ± 0.04	358,000
d-Ala	189 ± 5.2	2.7 ± 0.3	69,900
d-Glu	90.7 ± 6.6	12.1 ± 2.9	7,490
d-Arg	45.6 ± 4.5	19.2 ± 4.3	2,380

The enzyme activity was measured using the HRP-coupled method with 0.5–150 mM each amino acid at 55 °C. Each data represents the mean ± SD of triplicate measurements.

### Effect of pH, temperature, protein concentration, and FAD

**Figure 5:**
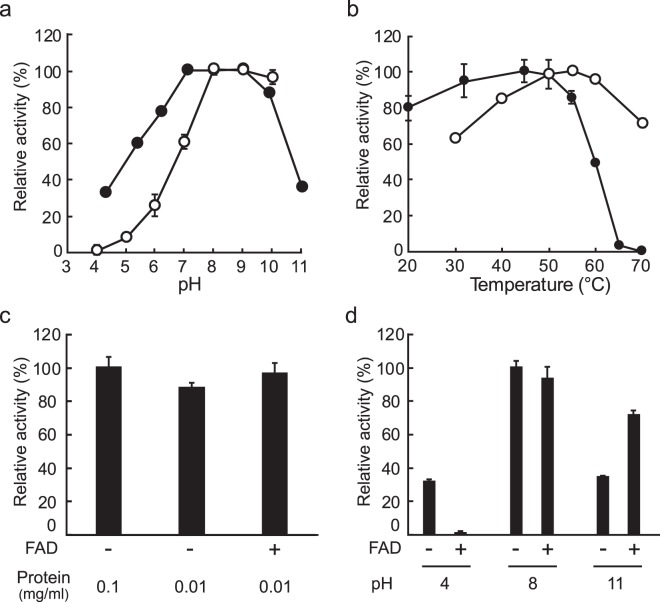
Effect of pH, temperature, protein concentration, and FAD. (**a**) Effect of pH on activity (open circles) and stability (closed circles). (**b**) Effect of temperature on activity (open circles) and stability (closed circles). (**c)** Effect of protein concentration and exogenous FAD on thermal stability. (**d**) Effect of exogenous FAD on pH stability. For the determination of stability, the residual activity was determined after incubation at 45 °C for 1 h in the presence and absence of exogenous 0.1 mM FAD. Each data point represents the mean ± the standard deviation of three measurements.

The enzyme exhibited higher activity at basic pHs with the highest at pH 8.0, but markedly lower activity at neutral and acidic pHs (e.g., <60% of that at pH 8.0) (Fig. 5a). The enzyme was stable between pH 6.0 and pH 10 for 1 h, where >80% of the activity at pH 8.0 was remained. The enzyme exhibited a higher activity at 50 –60 °C, with the highest at 55 °C (Fig. 5b). It was stable up to 50 °C for 1 h but gradually inactivated at >55 °C, and its activity was completely lost at 65 °C. *T*_50_ (the temperature at which it loses 50% activity) was ~60 °C. The thermal stability was only slightly decreased (approximately 12%) with decreasing the protein concentration from 0.1 mg/ml to 0.01 mg/ml, and the addition of exogenous FAD increased the stability at the lower protein concentration (Fig. 5c). In the case of the effect of FAD on pH stability, the addition of exogenous FAD significantly stabilized the enzyme at an alkaline pH but, strangely, conversely destabilized at an acidic pH (Fig. 5d).

### Effect of DAAO and DDO competitive inhibitors

The effect of DAAO and DDO competitive inhibitors on ReDAAO activity was analyzed (Supplementary Table 3). With 10 mM competitive inhibitors, the enzyme was most inhibited by the DDO inhibitor, d-malate, but only by 13.5%. The other competitive inhibitors decreased the activity by <9%. With 50 mM competitive inhibitors, the activity was significantly inhibited by DAAO inhibitors and one DDO inhibitor as follows: benzoate, crotonate, and d-malate decreased the activity by 13.2%, 22.6%, and 17.2%, respectively. These results showed that ReDAAO is sensitive to both DAAO and DDO inhibitors.

## Discussion

DAAO is a valuable enzyme for various practical applications, such as determining and quantifying d-amino acids and the production of materials for semisynthetic cephalosporins; therefore, the enzyme having high stability, high catalytic activity, and broad substrate specificity is desired. In this study, we isolated a strain of the thermophilic fungus *R*. *emersonii* that can efficiently grow on several d-amino acids and further isolated the fungal DAAO gene. The recombinant DAAO exhibited unique broad substrate specificity (i.e., the enzyme efficiently catalyzed the reaction of an unusual substrate, d-Glu and CPC) and high thermal stability as well as high catalytic activity. These results showed that ReDAAO has valuable and unique properties that are useful for various practical applications.

To isolate a practically useful DAAO which has high thermal stability, high catalytic activity, and broad substrate specificity, we first aimed to isolate the thermophilic fungi that can grow well on d-Asn, d-Gln, and d-His as the sole source of nitrogen source, because the practically useful fungal DAAOs, RgDAAO and TvDAAO, which have high catalytic activity and broad substrate specificity and can be used in the production of 7-ACA from CPC, can efficiently act on these d-amino acids^16^. Additionally, to our knowledge, no other enzymes, except DAAO, have been reported to accept d-Asn, d-Gln, and d-His as substrates in fungi^26^; therefore, the growth of the thermophilic fungi on the d-amino acids was predicted to be supported mainly by DAAO, as shown in other fungal DAAOs^27^. However, the growth level of *R*. *emersonii* strain YA on each d-amino acid appeared to be not fully comparable to the respective activities of ReDAAO (Fig. 1, Table 1). Other factors, such as the different uptake efficiencies among the d-amino acids, might also affect the growth on these d-amino acids. Nevertheless, as we successfully obtained a targeted DAAO, other novel fungal DAAOs that have unique and useful substrate specificities could be obtained by the screening based on the growth of fungi on d-amino acids.

The optimal pH of ReDAAO was in alkaline region similar to those of RgDAAO and pkDAAO but not to that of TvDAAO (Fig. 5a), which has the optimal pH within the neutral region^17,28^. Additionally, the pH stability of ReDAAO was somewhat different from that of the fungal DAAOs, RgDAAO and TvDAAO: The fungal DAAOs are stable at pH 6.0, while ReDAAO was slightly unstable at the pH. ReDAAO had high catalytic activity at 55 °C, which was comparable to that of other fungal DAAOs and higher than mammalian and bacterial DAAOs, although the assay temperature in this study was different from that in the assay of other DAAOs. Mammalian DAAOs typically have a long loop covering the active site, called active-site lid, that opens and closes to accept substrates and to release products^29^. This conformational change is slow and thereby lowers the overall turnover rate^30^. The fungal RgDAAO lacks this lid, which has been considered to be the reason for its higher catalytic activity^24^. In pig kidney DAAO (pkDAAO), the active site lid is formed by 13 amino acid residues, Thr216–Tyr228 (Fig. 2). In the corresponding region of ReDAAO as well as RgDAAO, a relatively small number of amino acid residues (10 and 9 amino acid residues, respectively) were found in the sequence alignment, suggesting the possibility that ReDAAO might also lack the lid; however, the same number of amino acid residues as ReDAAO was also observed in the corresponding lid region of the low catalytic bacterial RxDAAO, while a comparable or a larger number of residues than that of mammalian DAAOs were found in the lid region of the higher catalytic TvDAAO (12 amino acid residues) and DDO of the yeast *Cryptococcus humicola* (15 amino acid residues) (Fig. 2). Thus, further studies, such as those on determining their crystal structures, might be necessary to clarify the relationship between the catalytic turnover rate and the loop length.

**Figure 6:**
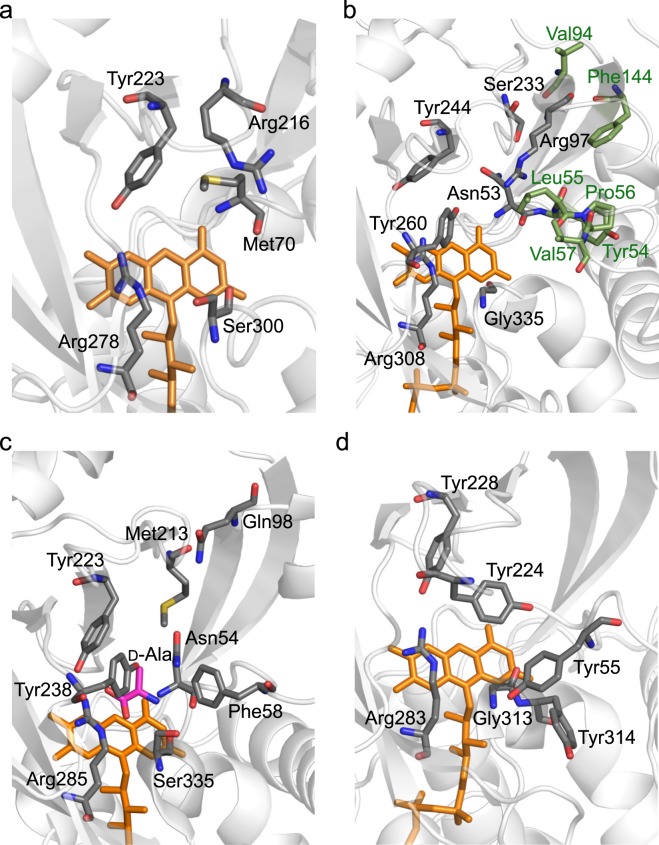
Active site structures of ReDAAO, RgDAAO, pkDAAO, and mouse DDO. Oxygen, nitrogen, and sulfur atoms are shown in red, blue, and yellow, respectively. Carbon atoms of the amino acid residues of proteins and substrates are shown in gray or green and pink, respectively. FAD is shown in orange. (**a**) Active site model of mouse DDO. (**b**) Active site model of ReDAAO. The hydrophobic amino acid residues covering the side chain of substrates are indicated by green. (**c**) Active site of RgDAAO crystal structure (PDB accession no. 1c0p). (**d**) Active site of pkDAAO crystal structure (PDB accession no. 1kif). Each image was rendered using PyMOL (https://pymol.org/2/).

ReDAAO accommodated various d-amino acids and CPC as substrates, similar to the fungal RgDAAO and TvDAAO, but there were some differences in their substrate use (Table 1). RgDAAO showed relatively moderate activity against d-Trp and d-Pro, whereas ReDAAO showed much lower activity against these d-amino acids. Conversely, RgDAAO showed much lower activity against d-Arg, whereas, as with TvDAAO, ReDAAO showed moderate activity against the basic d-amino acid^25,31^. The most intriguing feature of ReDAAO was its high activity against d-Glu, which is a typical substrate of DDO but not of DAAO (Table 1). The apparent *k*_cat_/*K*_m_ value for d-Glu of ReDAAO at an optimum temperature was approximately 600-fold higher than that of RgDAAO^25^. Such a DDO property of ReDAAO was also confirmed in its sensitivity against DDO competitive inhibitors (Supplementary Table 3). These results suggested that ReDAAO might be a novel DAAO having both DAAO and DDO activities. The amino acid residues located at the upper side of the active site, including those of the active site lid, are involved in the substrate specificity of DAAO^25,32^. In RgDAAO, Met213 in the active site plays a most important role in the substrate specificity, and the mutation of Met213 into Arg residue gave the oxidase activity against d-Asp^25^, a general substrate of DDO. In the spatially corresponding position of ReDAAO model, Ser233 is likely to be located instead of Ile232 that was predicted by amino acid sequence alignment described above (Figs 2 and 6b), suggesting that Ser233 might be involved in the substrate specificity. In addition, the substitution of a part of the lid of pkDAAO with the corresponding part of human DDO, which contains an Arg residue, also provided DDO activity^32^. In mouse DDO, Arg216 in the active site is vital for accepting acidic d-amino acids as substrates by possibly interacting with the β-carboxy side chain of the substrates (Fig. 6a)^33^. In the model structure of ReDAAO, an Arg residue at position 97 was found at the upper side of the active site (Fig. 6b); whereas, in RgDAAO, a Gln residue is present in the corresponding spatial position (Fig. 6c), which suggested the possibility that Arg97 might play an important role in the higher activity against d-Glu.

ReDAAO exhibited higher activity, in particular, toward hydrophobic d-amino acids (Table 1). In the active site of ReDAAO model, the hydrophobic amino acid residues Tyr54, Leu55, Pro56, Val57, Val94, and Phe144 are located at the position covering the side chain of substrates (Fig. 6b). In mammalian DAAOs, the corresponding hydrophobic region, mainly formed by the amino acid residues around Tyr55 with Tyr314 in pkDAAO (Figs 2 and 6d), is called the hydrophobic secondary binding pocket and, besides the active site lid, play an important role in the substrate specificity, activity, product egress, and inhibitor binding^34,35^. The van der Waals and hydrophobic interactions in this region of ReDAAO might also contribute to the higher activity toward hydrophobic substrates and the substrate specificity. The importance of hydrophobic interactions could also be explained by considerably lower activities against d-Thr than that against d-Val and also against d-Asn than that against d-Leu (Table 1), which are similar in size and shape but different in hydrophobicity. In addition, the shape of the side chain of substrates might affect the activity, because the activities against d-Thr and d-*allo*-Thr as well as d-Ile and d-*allo*-Ile were largely different (Table 1). The substitutions of Phe54 of TvDAAO and Tyr55 of pkDAAO with other amino acid residues change their substrate specificities (Figs 2 and 6d)^36^. Val57 was likely to be placed at the corresponding position in ReDAAO (Figs 2 and 6b), suggesting that Val57 and also adjacent amino acid residues might cause the different activities toward the isomers possibly by their steric hindrance. Tyr55 of mammalian DAAOs, which are the corresponding amino acid residues of Val57 of ReDAAO, plays a role in controlling access the active site, together with Y224, Y228, and Y314 (https://doi.org/10.1371/journal.pone.0198990). The substitution of Tyr55 with a smaller amino acid residue Ala improve the activity toward bulky substrates such as d-Trp, which might be due to significantly improved access to the active site by the substitution. From these findings, we first supposed that the broad substrate specificity of ReDAAO was due to the smaller amino acid residue Val57 instead of a Tyr or Phe residue found at the corresponding position in other DAAOs (Fig. 2). However, as described above, ReDAAO was less active on d-Trp (Table 1), while RgDAAO, which has a Phe residue (Phe58 in Fig. 6c) at the corresponding position, is moderately active on the bulky substrate^37^. The further analysis, such as the determination of the crystal structure and mutational analysis, might be however needed to more clearly understand the structural factors that govern the substrate specificity.

The *T*_50_ of ReDAAO (60 °C) was much higher than those of mammalian and other fungal DAAOs, which are 44 °C, 39 °C, and 54 °C for pkDAAO, RgDAAO, and TvDAAO, respectively, at 0.1 mg/ml protein concentration and after incubating for 30 min^28^, and comparable to that of the thermophilic bacterial RxDAAO (64 °C) at 0.1 mg/ml protein concentration and after incubating for 1 h^17^. These comparisons showed that ReDAAO has much higher thermal stability than known DAAOs, except for thermophilic bacterial RxDAAO. FAD and subunit interactions have been shown to affect the thermal stability of DAAO^17,38,39^. In this study, ReDAAO was more thermally stable in the presence of an excess amount of exogenous FAD (Fig. 5c), suggesting that the interaction with FAD might be involved in the higher thermal stability. In the effect of exogenous FAD on pH stability, we obtained an intriguing result that exogenous FAD considerably destabilized ReDAAO at an acidic condition (Fig. 5d). As the enzyme was stabilized by exogenous FAD at an alkaline condition, the protonation of exogenous FAD could cause the decreased stability at the acidic condition. Because hydrogen bonds are important for binding to FAD^40^, the protonation of FAD might impair the hydrogen-bonding interactions, leading to the enhanced destabilization of the enzyme. Additionally, as ReDAAO was a tetramer, the subunit interaction might be also involved in the higher thermal stability. The thermal stability of DAAOs is usually depended on the protein concentration: the decrease in protein concentration markedly decreased the thermal stability due to subunit dissociation^41^. However, the thermal stability of ReDAAO was only slightly decreased by decreasing protein concentration (Fig. 5c). As the tetramer formation of ReDAAO was still observed at the lower protein concentration, tight subunit interactions might also contribute to the higher thermal stability. It has been also reported that the oxidation of Cys108 in TvDAAO, which is not located in the active site, significantly decreases the stability by affecting the protein environment of the bound FAD^42^. In the corresponding position of ReDAAO, a Cys residue was not observed (Fig. 2), suggesting the lack of destabilizing Cys residue might be also a reason for the higher stability.

## Methods

### Materials

d-Leu and d-His were purchased from Wako Pure Chemical Industries (Osaka, Japan), and d-Ile was from the Peptide Institute (Osaka, Japan). d-Asp was a gift from Tanabe Pharmaceutical (Osaka, Japan). All other d-amino acids were purchased from Nacalai Tesque (Kyoto, Japan). DNA modification enzymes were obtained from Toyobo (Osaka, Japan) or Takara Bio (Shiga, Japan). All other chemicals were of analytical reagent grade and were from Wako Pure Chemical Industries, Nacalai Tesque, or Sigma-Aldrich (St. Louis, MO, USA). Primers were synthesized by Eurofins (Tokyo, Japan). Composts were purchased from local markets.

### Microbial strains and cultivation media

*E*. *coli* strain DH5α and strain BL21(DE3) pLysS were used as the host for DNA manipulation and protein expression, respectively. Thermophilic fungi were grown on potato dextrose agar (PDA) medium (Merck KGaA, Darmstadt, Germany). *E*. *coli* cells were cultivated in LB. For solid media, 2.0% or 1.5% (w/v) agar was added to the media for fungi and *E*. *coli*, respectively.

### Isolation of thermophilic fungi

One gram of compost was suspended in 10 ml saline solution. An aliquot of the suspensions was spread onto PDA medium containing 0.5 g/l chloramphenicol and 0.05 g/l gentamicin and incubated at 50 °C and 60 °C for 2 weeks.

### D-Amino acid-assimilating ability of thermophilic fungi

Thermophilic fungi were inoculated onto synthetic agar media composed of a yeast carbon base (Becton Dickinson Co., Franklin Lakes, NJ, USA) containing 50 mM d-Ala, d-Asn, d-Gln, or d-His as the sole nitrogen source and incubated at 50 °C for 1 week.

### Identification of thermophilic fungi

The genomic DNA of thermophilic fungi was isolated as described by Yu *et al*.^43^. The internal transcribed spacer (ITS) region was amplified using Tks Gflex DNA polymerase with the primers ITS1 (5′-TCCGTAGGTGAACCTGCGG-3′) and ITS4 (5′-TCCTCCGCTTATTGATATGC-3′)^44^ and genomic DNA as a template (Takara Bio, Shiga, Japan). The reaction mixture was preheated at 98 °C for 1 min, followed by 30 cycles at 98 °C for 10 s, 55 °C for 10 s, and 68 °C for 15 s, with a final extension at 68 °C for 10 min. The product was ligated into the pGEM-T Easy vector (Promega, Madison, WI, USA) and sequenced. NCBI-BLAST was used to conduct the homology search of the ITS region of strain YA. A multiple sequence alignment of the ITS region was conducted using T-Coffee (https://tcoffee.vital-it.ch/apps/tcoffee/do:regular) and was edited using Gblocks v. 0.91b (http://molevol.cmima.csic.es/castresana/Gblocks_server.html). A phylogenetic tree was constructed using the maximum likelihood method with MEGA 7.0 (https://www.megasoftware.net/home).

### Isolation DAAO homologous gene of *R*. *emersonii s*train YA

The DAAO homologous gene (*T310_5354*) of *R*. *emersonii* strain CBS 393.64 was identified by a BLAST search using amino acid sequences of RgDAAO and TvDAAO in the fungal genome database Ensembl Fungi (http://fungi.ensembl.org/). The corresponding homologous gene of strain YA (*ReDAAO*) was amplified using Tks Gflex DNA polymerase with the primers ReDAAO-F (5′-ATGGCAACCAATAACATCG-3′) and ReDAAO-R (5′-TTACAGCCTCGCCTCTCC-3′) and the genomic DNA of strain YA as a template. The reaction mixture was preheated at 98 °C for 1 min, followed by 30 cycles at 98 °C for 10 s, 50 °C for 15 s, and 68 °C for 84 s, with a final extension at 68 °C for 10 min. The resulting product was inserted into the pGEM-T Easy vector and sequenced. *ReDAAO* ORF was synthesized based on the codon usage of *E*. *coli* by GenScript (Piscataway, NJ, USA) and ligated into pET15b.

### Expression and purification of ReDAAO in *E*. *coli*

*E*. *coli* BL21(DE3) pLysS harboring *ReDAAO* was precultivated in LB medium containing 100 µg/ml ampicillin and 34 µg/ml chloramphenicol at 37 °C for 14 h. One milliliter of preculture was transferred to 200 ml LB medium containing the same antibiotics and cultivated at 37 °C. When the optical density at 660 nm reached approximately 0.5, isopropyl β-d-thiogalactopyranoside (IPTG) was added to the medium at a final concentration of 0.1 mM, and the cells were further cultivated at 37 °C for 4 h. The cells were collected after centrifuging at 5,000 × *g* for 10 min at 4 °C and were resuspended in 50 mM potassium phosphate (KPi) buffer (pH 8.0). The cells were sonicated 25 times for 30 s with the UD-201 sonicator (Tomy Seiko, Tokyo, Japan) at an output of 10 and a duty cycle of 50%. The homogenate was centrifuged at 20,000 × *g* for 30 min at 4 °C, and the supernatant was filtrated through a 0.45-µm membrane filter (Omnipore; Millipore, Billerica, MA, USA). After adding imidazole at a final concentration of 10 mM, the supernatant was applied to a TALON metal-affinity column (Clontech, Mountain View, CA, USA). The column was washed once with 50 mM KPi buffer (pH 8.0) containing 300 mM NaCl and 30 mM imidazole, and the bound proteins were eluted with the same buffer containing 150 mM imidazole. The eluate was concentrated using an Amicon Ultra-15 centrifugal filter device with a 10 K molecular–weight cutoff membrane (Millipore) and dialyzed overnight against 50 mM KPi buffer (pH 8.0) containing 5% (v/v) glycerol.

### SDS-PAGE and molecular determination

The homogeneity of purified ReDAAO was determined using 12.5% SDS-PAGE. The native molecular mass was determined by gel filtration on a Superdex 200 HR 10/30 column (GE Healthcare, Tokyo, Japan) equilibrated with 50 mM KPi buffer (pH 8.0) containing 150 mM NaCl and 5% (v/v) glycerol at a flow rate of 0.3 ml/min. The molecular mass standards used were catalase (232 kDa), aldolase (158 kDa), conalbumin (75.0 kDa), chymotrypsinogen A (25.0 kDa), and RNase A (13.7 kDa).

### Enzyme assay and protein determination

Enzyme activity was determined using a horseradish peroxidase (HRP)–coupled reaction with phenol and 4-aminoantipyrine (4-AAP) or using 2,4-dinitrophenylhydrazine (DNPH). The HRP-coupled reaction mixture contained 20 mM amino acid (2 mM d-Tyr), 2 mM phenol, 1.5 mM 4-AA, 2.5 U highly stabilized HRP (Sigma-Aldrich), and 0.02 mM FAD in 50 mM KPi buffer (pH 8.0). The enzyme activity was determined at 505 nm using a molar extinction coefficient for quinonimine of 5.86 × 10^3^ M^−1^ cm^−1^ ^45^. A DNPH assay was conducted as described previously^17^. The protein concentration was determined using the BioRad protein assay kit (Bio-Rad, Hercules, CA, USA) with fetal bovine serum albumin as a standard.

### Substrate specificity and kinetic parameters

The substrate specificity and the apparent kinetic parameters were determined using the HRP-coupled method at 37 °C and 55 °C, respectively. For determining the apparent kinetic parameters, enzyme assays were conducted with 0.5–100 mM d-amino acids. The apparent kinetic parameters were determined by fitting the Michaelis–Menten equation to the initial reaction rates using SigmaPlot 12.5 (Systat Software, San Jose, CA, USA).

### Absorption spectra

The absorption spectra of the enzyme (0.16 mg/ml) in 50 mM KPi buffer (pH 8.0) were measured before and after incubation with 50 mM d-Val at 40 °C for 10 min using a Shimadzu UV-3100PC spectrophotometer (Kyoto, Japan).

### Thin-layer chromatography

ReDAAO cofactor was analyzed by thin-layer chromatography (TLC) as described by Yamada *et al*.^46^. The purified enzyme (0.185 mM) in 50 mM KPi buffer (pH 8.0) containing 5% (v/v) glycerol was incubated at 100 °C for 10 min in the dark. After centrifugation at 20,000 × *g* at 4 °C for 30 min, the supernatant (1.0 µl) was subjected to TLC on silica gel 60 F-254 plates (2-mm thickness; Merck) and developed in 5% Na_2_HPO_4_ (w/v) as the solvent phase. FAD and flavin mononucleotide (FMN; 1 µl, 0.1 mM each) in 50 mM KPi buffer (pH 8.0) containing 5% (v/v) glycerol were used as the standards and monitored under UV light (312 nm).

### Effect of temperature, pH, protein concentration, and FAD

The optimum pH was determined by measuring the activity at different pH values in 50 mM GTA buffer (16.7 mM 3,3-dimethyl glutaric acid, 16.7 mM tris, and 16.7 mM 2-amino-2-methyl-1,3-propanediol). The optimum temperature was determined by measuring the activity at different temperatures. The pH stability was determined by measuring the residual activity after incubating 0.1 mg/ml enzyme at 45 °C for 1 h in 50 mM GTA buffer at different pH values. The thermal stability was determined by measuring the residual activity after incubating 0.1 mg/ml enzyme in 50 mM KPi buffer (pH 8.0) for 1 h at different temperatures. The effect of protein concentration and the effect of exogenous 0.1 mM FAD on thermal stability or pH stability were analyzed after incubation at 45 °C for 1 h. The enzyme assays for pH and thermal stability were conducted using the HRP-coupled method at 37 °C with 20 mM d-Val as a substrate, and those for optimum pH and temperature were conducted using the DNPH method at 55 °C with 20 mM d-Val as a substrate.

### Effect of competitive inhibitors

The effects of competitive inhibitors were analyzed by assaying the activity in the presence of 10 mM or 50 mM each inhibitor. These assays were conducted using the DNPH method at 55 °C with 20 mM d-Val as a substrate.

### Construction of three-dimensional model structures

The three-dimensional model structures of ReDAAO and mouse DDO were generated using I-TASSER Server (http://zhanglab.ccmb.med.umich.edu/I-TASSER/) and evaluated using PROCHECK^47^.

### Nucleotide sequence accession numbers

The DNA sequences of the ITS region of strain YA, the genomic DNA with introns, and the synthesized ORF DNA of *ReDAAO* gene were deposited in DDBJ/EMBL/GenBank under accession numbers LC436776, LC436777, and LC437087, respectively.

## Supplementary information

Supplementary information
