# Supplementary material for: A novel thermostable d-amino acid oxidase of the thermophilic fungus Rasamsonia emersonii strain YA
Source: Sci Rep. 2019 Aug 16;9:11948. doi: 10.1038/s41598-019-48480-y (PMC6697736; doi:10.1038/s41598-019-48480-y)
Supplement: Supplementary file 1 — Supplementary information [file 41598_2019_48480_MOESM1_ESM.pdf]

Supplementary information

A novel thermostable D-amino acid oxidase of the thermophilic fungus *Rasamsonia emersonii* strain YA

Yuya Shimekake[1], Takehiro Furuichi[1], Katsumasa Abe[1], Yoshio Kera[1], Shouji Takahashi[1,*]

[1]Department of Bioengineering, Nagaoka University of Technology, Nagaoka, Niigata 940-2188, Japan

[*]corresponding. shoutaka@vos.nagaokaut.ac.jp

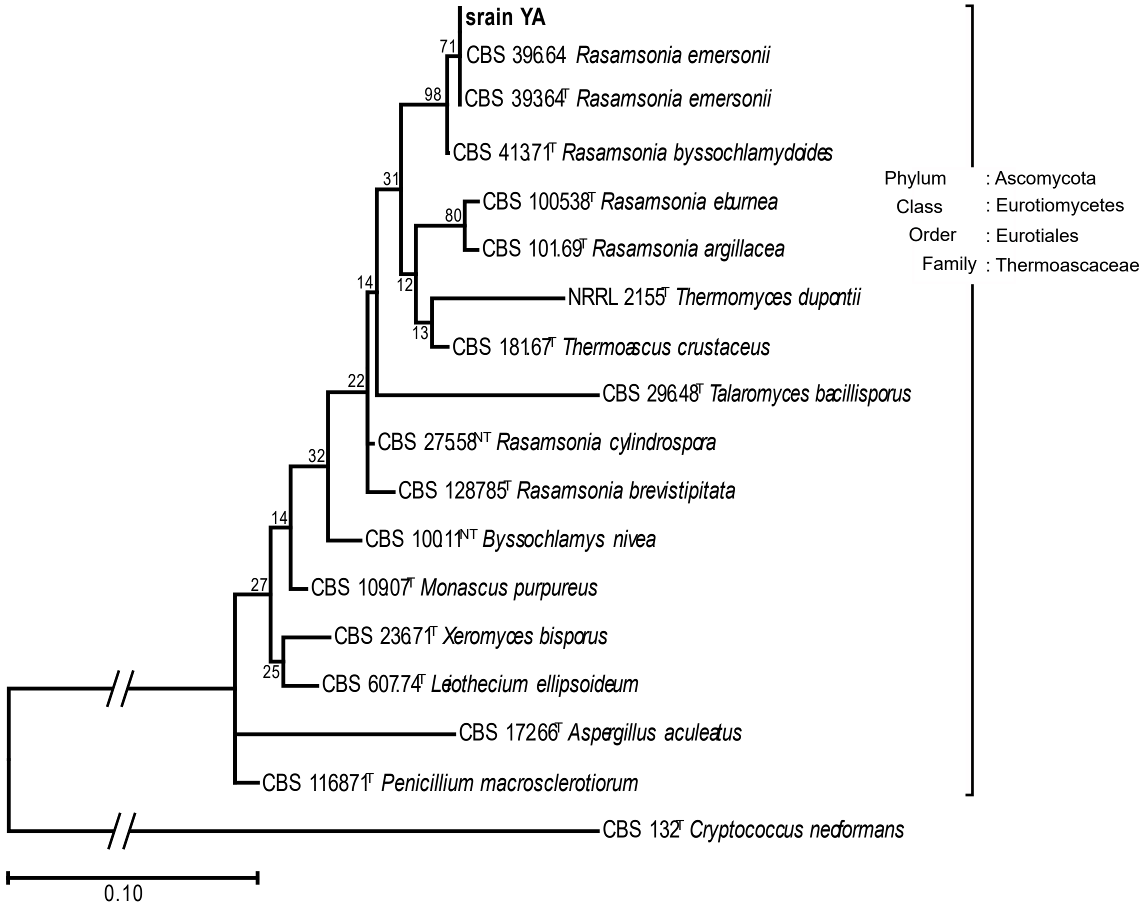

Supplementary Figure 1. A Phylogenetic tree based on ITS region sequence of YA and other fungi. The phylogenetic analysis was performed using the Maximum Likelihood method of MEGA 7.0 with 1,000 bootstrap trials. The numbers at nodes show bootstrap value percentages. The accession numbers of the ITS region sequences used for the analysis are as follows: *R. emersonii* CBS 396.64 (GenBank accession no. JF417479), *R. emersonii* CBS 393.64 (GenBank accession no. JF417478), *R. byssochlamydoides* CBS 413.71 (GenBank accession no. JF417476), *R. eburnean* CBS 100538 (GenBank accession no. JF417483), *R. argillacea* CBS 101.69 (GenBank accession no. JF417491), *T. dupontii* NRR 2155 (GenBank accession no. JF412001), *T. crustaceus* (GenBank accession no. JF412002), *T. bacillisporus* CBS 296.48 (GenBank accession no. JN899329), *R. cylindrospora* CBS 275.58 (GenBank accession no. JF417470), *R. brevistipitata* CBS 128785 (GenBank accession no. JF417488), *R. brevistipitata* CBS 128785 (GenBank accession no. JF417488), *B. nivea* CBS 100.11 (GenBank accession no. FJ389934), *M. purpureus* CBS 109.07 (GenBank accession no. KY635851), *X. bisporus* CBS 236.71 (GenBank accession no. KY635854), *L. ellipsoideum* CBS 607.74 (GenBank accession no. KF732839), *A. aculeatus* CBS 172.66 (GenBank accession no. FJ629320) and *P. macrosclerotiorum* CBS 116871 (GenBank accession no. KJ834511), and *C. neoformans* CBS 132 (GenBank accession no. AF444326) was used as a outgroup. The superscript T and NT shows type and neotype strain, respectively.

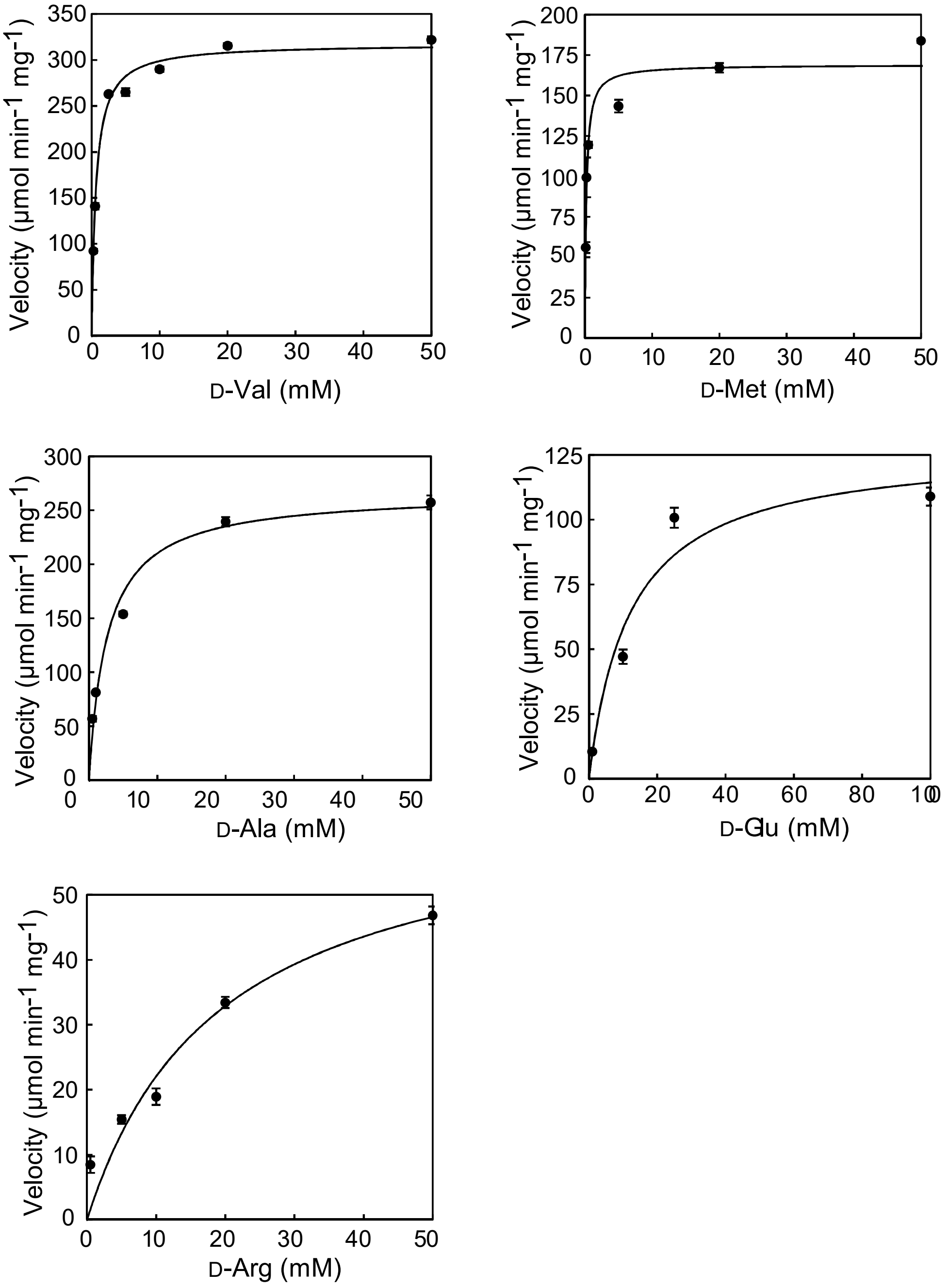

Supplementary Figure 2. Kinetic analysis of ReDAO. The kinetic analysis was performed in 50 mM KPi buffer (pH 8.0) for each D-amino acid at 55°C using a HRP-coupled method. The fitting and the plotting were performed using the program SigmaPlot 12.5. Each data point represents the mean ± SD of three measurements.

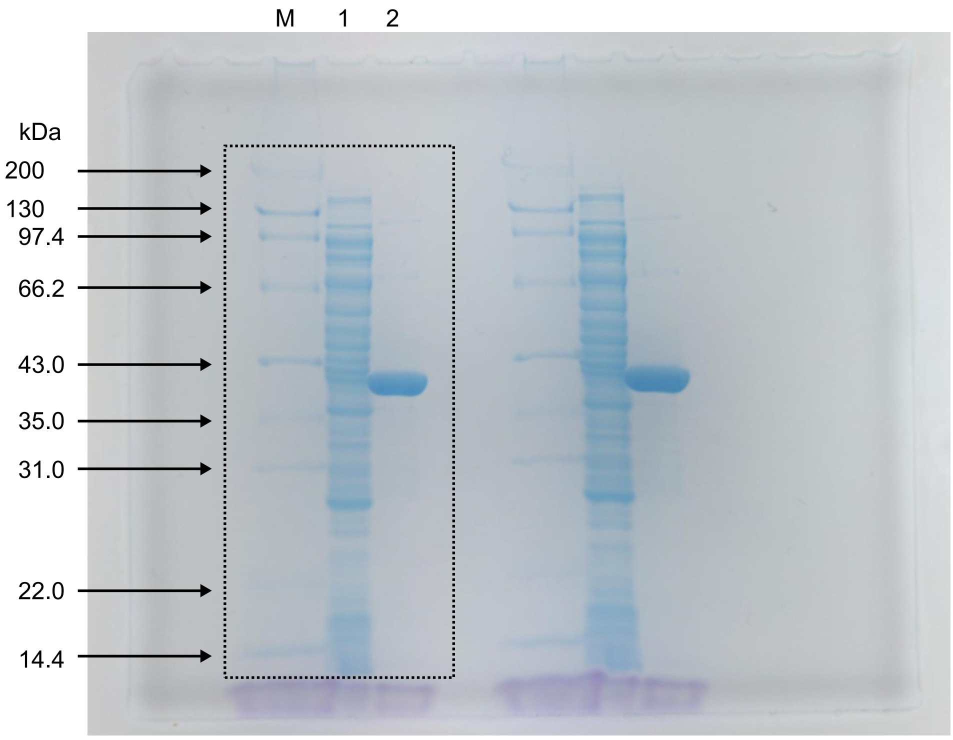

Supplementary Figure 3. The original SDS-PAGE image displayed on Figure 3.
Lanes: M, protein marker; 1, crude extract; 2, purified ReDAO.

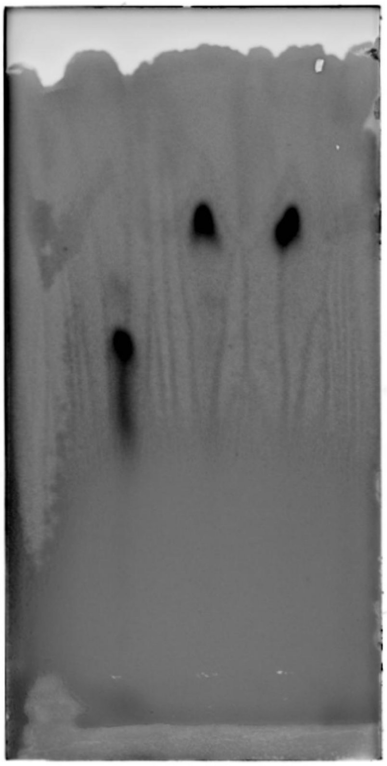

Supplementary Figure 4. The original TLC image displayed on Figure 3b.
Lanes: 1. 0.1 mM FMN; 2, 0.1 mM FAD; 3, flavin from 0.185 mM ReDAO.

Supplementary Table 1. Substrate specificity of the crude extract of *E. coli* cells expressing ReDAO gene

Substrates	Mean relative activity (%) ± SD
D-Ile	100 ± 22
D-Val	94.9 ± 25.3
D-Met	83.2 ± 3.8
D-Thr	76.2 ± 18.4
D-Phe	69.8 ± 6.2
D-Ala	67.3 ± 16.8
D-Ser	64.7 ± 4.6
D-Gln	62.2 ± 15.2
D-Leu	60.8 ± 9.5
D-Glu	55.5 ± 12.3
D-Arg	54.7 ± 12.5
D-Trp	52.3 ± 8.0
D-Pro	46.5 ± 19.3
D-Tyr	46.8 ± 13.0
D-Asn	37.5 ± 3.5
D-His	16.7 ± 5.8
D-Lys	21.1 ± 4.7
D-Asp	5.6 ± 0.9

The enzyme activity was measured using the HRP-coupled method with 20 mM each amino acid except 2 mM D-Tyr at 37°C. Each data represents the mean ± SD of triplicate measurements.

Supplementary Table 2. Purification of ReDAO expressed in *E. coli*

Step	Total protein (mg)	Total activity (U)	Specific activity (U/mg)	Purification (fold)	Yield (%)
Crude extract	137	389	2.84 ± 0.03	1	100
TALON Superflow	1.43	100	70.0 ± 1.0 (37°C)<br>112 ± 5.0 (55°C)	24.6	25.7

The enzyme activity was measured using the HRP-coupled method with 20 mM D-Ile at 37°C and 55°C. Each data represents the mean ± SD of triplicate measurements. The starting material was 4.0 g of *E. coli* cell paste from a 600 ml fermentation.

Supplementary Table 3. Effect of DAAO and DDO competitive inhibitors on the activity of purified ReDAAO

Compounds	Concentration (mM)	Relative activity
Inhibitors		
None		100
DAAO inhibitors		
Benzoate	10	93.5 ± 1.4
	50	86.8 ± 6.5
Anthranilate	10	95.7 ± 3.4
	50	103 ± 1.8
Crotonate	10	101 ± 0.8
	50	77.4 ± 2.2
DDO inhibitors		
Malonate	10	91.5 ± 4.5
	50	98.6 ± 3.1
meso-Tartrate	10	96.5 ± 2.7

	50	93.5 ± 5.7
D-Malate	10	86.5 ± 2.8
	50	82.8 ± 1.3

For DAAO and DDO competitive inhibitors, the enzymatic activity was assayed using the DNPH method in the presence of 10 or 50 mM each compound at 55°C with 20 mM D-Val as a substrate. Each data represents the mean ± SD of triplicate measurements.
